# Removal of a Fibro-Cellular Tumor of the Scrotum

**Published:** 1864-07

**Authors:** A. J. Baxter

**Affiliations:** Chicago


### REMOVAL OF
### A FIBRO-CELLULAR TUMOR OF THE SCROTUM.

By A. J. BAXTER, M. D., of Chicago.

The rarity of fibro-cellular tumors, properly so-called, in comparison with outgrowths of the same texture; and the very early age at which the one about to be described, occurred have induced me to give it publicity.

Notwithstanding the extensive observations of Mr. Paget, he has seen but two specimens of the variety of tumor under consideration, in the scrotum—both of which occurred in subjects above seventy years of age.

Charles Vanduren, a Swede, aged 13, delicate, of a sympathetic temperament, consulted me, May 2d, in regard to a "lump in his bag." Upon examination I found a tumor situated in the right side of the scrotum, slightly pendulous, which was first noticed about three years since. In volume it was larger than two fists, slightly lobulated, of an elastic feel, though of equal density, and conveying the idea of cysts. Several enlarged veins ramified over its cutaneous surface, skin healthy in appearance and density, freely movable over the subjacent parts, though the *tumor* seemed to be firmly fixed, deep in the perineum, which was found to be the case during the operation for its removal.

The patient did not pay much attention to it during the first two years, as it was not larger than an English walnut, and has never been a source of pain; but during the last year it grew very rapidly, and continued to do so up to the time of extirpation. No enlargement of the inguinal glands.

The patient being chloroformed, the operation was commenced by making an incision from near the root of the penis and on the right of the raphe, to the perineum, which freely exposed the tumor contained in a loose capsule. The testicle was pushed high up, the tunica vaginalis testis was distended with a transparent fluid, and adherent to the tumor. No trouble was encountered in its removal, the adhesions to the tunica vaginalis were readily broken up by the handle of the scalpel. One artery, probably as large as the radial, passed into the posterior or perineal portion of the tumor, which was felt and ligated before being divided. The weight of the tumor a couple of hours after removal was three-quarters of a pound. A portion of the tumor was examined under the microscope, and so fully coincided with the description given by Mr. Paget, that I beg leave to refer to his article on the subject. The patient is now fully recovered.

